# Prevalence of Dural Ectasia in Loeys-Dietz Syndrome: Comparison with Marfan Syndrome and Normal Controls

**DOI:** 10.1371/journal.pone.0075264

**Published:** 2013-09-25

**Authors:** Atsushi K. Kono, Masahiro Higashi, Hiroko Morisaki, Takayuki Morisaki, Hiroaki Naito, Kazuro Sugimura

**Affiliations:** 1 Department of Radiology, Kobe University Graduate School of Medicine, Kobe Japan; 2 Department of Radiology, National Cardiovascular Research Center, Suita, Japan; 3 Department of Bioscience, National Cardiovascular Research Center, Suita, Japan; Pasteur Institute of Lille, France

## Abstract

**Background and Purpose:**

Dural ectasia is well recognized in Marfan syndrome (MFS) as one of the major diagnostic criteria, but the exact prevalence of dural ectasia is still unknown in Loeys−Dietz syndrome (LDS), which is a recently discovered connective tissue disease. In this study, we evaluated the prevalence of dural ectasia in LDS according by using qualitative and quantitative methods and compared our findings with those for with MFS and normal controls.

**Material and Methods:**

We retrospectively studied 10 LDS (6 males, 4 females, mean age 36.3 years) and 20 MFS cases (12 males, 8 females, mean age 37.1 years) and 20 controls (12 males, 8 females, mean age 36.1 years) both qualitatively and quantitatively using axial CT images and sagittal multi-planar reconstruction images of the lumbosacral region. For quantitative examination, we adopted two methods: method-1 (anteroposterior dural diameter of S1> L4) and method-2 (ratio of anteroposterior dural diameter/vertebral body diameter>cutoff values). The prevalence of dural ectasia among groups was compared by using Fisher’s exact test and the Tukey−Kramer test.

**Results:**

In LDS patients, the qualitative method showed 40% of dural ectasia, the quantitative method-1 50%, and the method-2 70%. In MFS patients, the corresponding prevalences were 50%, 75%, and 85%, and in controls, 0%, 0%, and 5%. Both LDS and MFS had a significantly wider dura than controls.

**Conclusions:**

While the prevalence of dural ectasia varied depending on differences in qualitative and quantitative methods, LDS as well as MFS, showed, regardless of method, a higher prevalence of dural ectasia than controls. This finding should help the differentiation of LDS from controls.

## Introduction

Loeys–Dietz syndrome (LDS) is a newly discovered connective tissue disease caused by mutations in the gene of transforming growth factor β receptor (TGFBR) -1 or -2 [1], [2]. The cardinal features of LDS consist of craniofacial features characterized by widely spaced eyes (orbital hypertelorism), bifid uvula and/or cleft palate, and cardiovascular diseases such as aortic root dilatation or aortic dissection, arterial tortuosity and aneurysms [2], [3]. LDS shares many of its clinical features with Marfan syndrome (MFS) [4], which is an autosomal dominant connective tissue disorder caused by mutations in the gene of fibrillin-1 (FBN-1) [5]. The main features of MFS occur in skeletal, ocular, and cardiovascular areas, but other organs can also be affected, including skin, lung, and dura [6].

While LDS and MFS show striking pleiotropism and clinical variability, no clinical criteria for LDS have as yet been established in contrast to MFS, for which detailed diagnostic criteria have been developed [6], [7]. The old ‘Ghent nosology’ for MFS classifies the clinical manifestations into major and minor criteria [6]. Dural ectasia (DE), which is also observed in neurofibromatosis type 1 and Ehlers–Danlos syndrome [6], [8], is, after aortic dilatation/dissection, the second most common major criterion for MFS [8], [9]. In the revised Ghent nosology, DE is no longer a critical criterion for MFS. As the finding of DE is used for the scoring of systemic features when the patients show aortic diseases, but do not have the ectopia lentis, the importance of DE remains in the new criteria. While the importance of DE is well recognized in MFS, only a few reports have dealt with the prevalence of DE in LDS [1], [10], [11]. DE usually occurs in connective tissues diseases, and occurs in the lumbar or sacral spine due to gravity. DE is characterized by widening of the spinal canal, posterior scalloping of the vertebral body, increased thinning of the cortex of pedicles and laminae, widening of the neural foramina or the presence of a meningocele [6]. While at present there is no standardized method for the diagnosis of DE, some qualitative [6], [8] and quantitative methods [9], have been reported.

To establish clinical diagnostic criteria for LDS, the prevalence of DE has to be analyzed. In this study, we used qualitative and quantitative methods to evaluate the prevalence of DE in LDS in comparison with that in MFS and controls.

## Materials and Methods

The institutional review board (National Cardiovascular Research Center, Japan) approved this retrospective case-controlled study and written informed consent to analyze clinical data and gene mutations was obtained from all LDS and MFS subjects. In addition, written informed consent for the CT examinations was obtained from all subjects. The institutional review board waived written informed consent from the normal controls because this was a retrospective study.

### Patient Population

The CT data in our institutional database was reviewed from June 2007 to July 2010. Ten LDS patients with an identified mutation in TGFBR (6 males, 4 females, mean age 36.3 years, range 20−54 years) were retrospectively reviewed from our institutional database which comprised about 20 LDS patients. These 10 patients had undergone CT examination in the lower abdominal and pelvic regions and were consecutively enrolled. Nine of them (90%) were in the post-operative stage (1 with aortic repair, 3 with valve replacement, and 5 with both). Reasons for hospitalization were aortic root dilatation in 4 patients and aortic dissection in 6 patients. Gene analysis showed 4 mutations in TGFBR-1 and 6 in TGFBR-2.

Twenty MFS patients with an identified mutation in FBN-1 (12 males, 8 females, mean age 37.1 years, range 20−56 years) were also reviewed. MFS patients who were matched to LDS patients in gender and age were randomly selected from our database, which comprises more than 100 MFS patients with mutation in FBN-1. Seventeen of the enrolled patients (85%) were in the post-operative stage (3 with aortic repair, 7 with valve replacement, and 7 with both). Reasons for hospitalization were aortic root dilatation in 11 patients, aortic dissection in 8 patients, and mitral valve regurgitation in 1 patient.

All LDS and MFS patients underwent clinical examinations including a physical examination and laboratory tests by a cardiovascular team. Initial and follow up CT examinations were performed in a clinical setting as described below. Genetic analysis was performed for all patients in the same manner as previously reported [11].

Twenty control subjects (12 males, 8 females, mean age 36.1 years, range 22−52 years) who were matched to LDS patients in gender and age were also randomly selected from our CT database. These subjects did not meet any of the major nor minor criteria of Ghent nosology. No gene analysis was performed for this group.

MFS patients and control subjects were matched to LDS in gender and age to avoid the need for adjusting the values of variables after they had been measured.

### Imaging and Measurements

All LDS and MFS patients underwent CT examination (including CT angiography) using 16- or 64-MSCT at initial diagnosis or clinical follow-up for evaluation of the vascular tree. The CT covered the area from the thorax to the pelvis. Control subjects underwent CT examination covering the lower abdominal and pelvic regions as indicated by clinical need. Axial CT images with 2 mm slice thickness were obtained from all subjects and used to reconstruct multi-planar reconstruction (MPR) images. All images were transferred to CT image server and could be read on a PACS (Picture Archiving and Communication System) viewer. Two radiologists (A.K.K. and M.H. with 9 and 19 years experience, respectively, in diagnostic radiology) reviewed the CT images (qualitative inspection, described below) blinded to the genetic diagnosis. And one of the two radiologists (A.K.K.) evaluated CT images using the two quantitative methods described below.

### Qualitative Inspection

DE is defined as widening of the spinal canal, posterior scalloping of the vertebral body, increased thinning of the cortex of pedicles and laminae, widening of the neural foramina or the presence of a meningocele [8],[14],[15]. The presence of an anterior sacral meningocele was diagnosed when herniation of the dural sac resulting from a defect in the anterior surface of the sacrum was seen [16]. Presence of a lateral meningocele was diagnosed when the nerve root sleeve was wide throughout the intervertebral foramen and ended in a pouch [17].

### Quantitative Inspection

#### Method-1 proposed by Ahn et al. [9] (Fig. 1)

**Figure 1:**
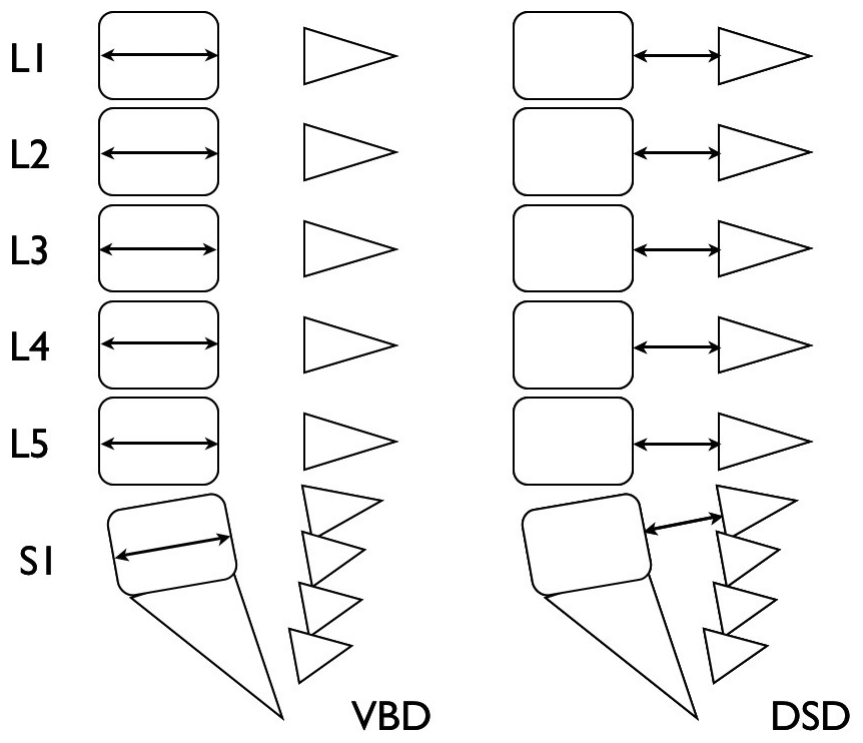
Scheme of sagittal CT images of the lumbosacral spine. Lines with arrows represent measurements of vertebral body diameters (VBD) and dural sac diameters (DSD).

Dural sac diameter (DSD) was measured in the mid-sagittal plane of the MPR image from the lumbus through to the sacrum. DE is diagnosed when the sagittal anteroposterior diameter of the spinal canal at S1 or below is greater than that at the mid axis of L4.
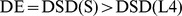



#### Method-2 proposed by Oosterhof et al. [12] (Fig. 1)

The vertebral body diameter (VBD) and DSD were measured perpendicular to the long axis of the dural sac and vertebral body. VBD and DSD values were obtained at the midcorpus level of L1 to S1. The dural sac ratio (DSR) was calculated at L1 to S1 by dividing DSD by VBD. DE is considered present if DSR exceeds the cutoff value, which is defined as mean+2 SD of controls.
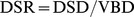


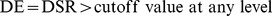



### Statistical Analysis

For the statistical analysis, JMP software (version 8.0, SAS Institute Inc., CA, USA) was used. Continuous data were expressed as mean±SD.

We evaluated and compared the prevalence of DE in three groups by using one qualitative and two quantitative methods. The two-tailed student *t* test was used to compare continuous variables and Fisher’s exact test for discrete variables. The DSRs among groups were evaluated with Tukey-Kramer test (alpha value of 0.05 was adopted). A p value <0.05 was considered statistically significant.

## Results

Patient characteristics are listed in Table 1. There were no differences in sex and gender among the three groups.

**Table 1:** Patient characteristics and prevalence of DE determined with qualitative and quantitative methods.

	LDS (n = 10)	MFS (n = 20)	Control (n = 20)	Difference*
Gene abnormality	TGFBR-1 or -2	FBN-1		
Gender (m:f)	6∶4	12∶8	12∶8	NS
Age (y)	36.3±12.6	37.1±11.2	36.1±8.6	NS
No. of DE identified with qualitative method				
DE-positive	4 (40)	16 (80)	0 (0)	a, (p = 0.04); b, (p = 0.0077); c, (p<0.0001)
No. of DE identified with method-1				
DE-positive	5 (50)	15 (75)	0 (0)	b, (p = 0.0018); c, (p<0.0001)
No. of DE identified with method-2				
DE-positive at any level	7 (70)	17 (85)	1 (5)	b, (p = 0.00068); c, (p<0.0001)
L1	4 (40)	3 (15)	1 (5)	
L2	3 (30)	3 (15)	0 (0)	
L3	3 (30)	3 (15)	0 (0)	
L4	0 (0)	4 (20)	0 (0)	
L5	2 (20)	7 (35)	1 (5)	
S1	6 (60)	16 (80)	0 (0)	

LDS, Loeys-Dietz syndrome; MFS, Marfan syndrome; DSR, dural sac ratio; DE, dural ectasia; NS, not significant; a, difference between LDS and MFS; b, difference between LDS and control; c, difference between MFS and control.

Difference*, In this column, p values are shown. Differences were not tested for each level from L1 to S1.

**Figure 2:**
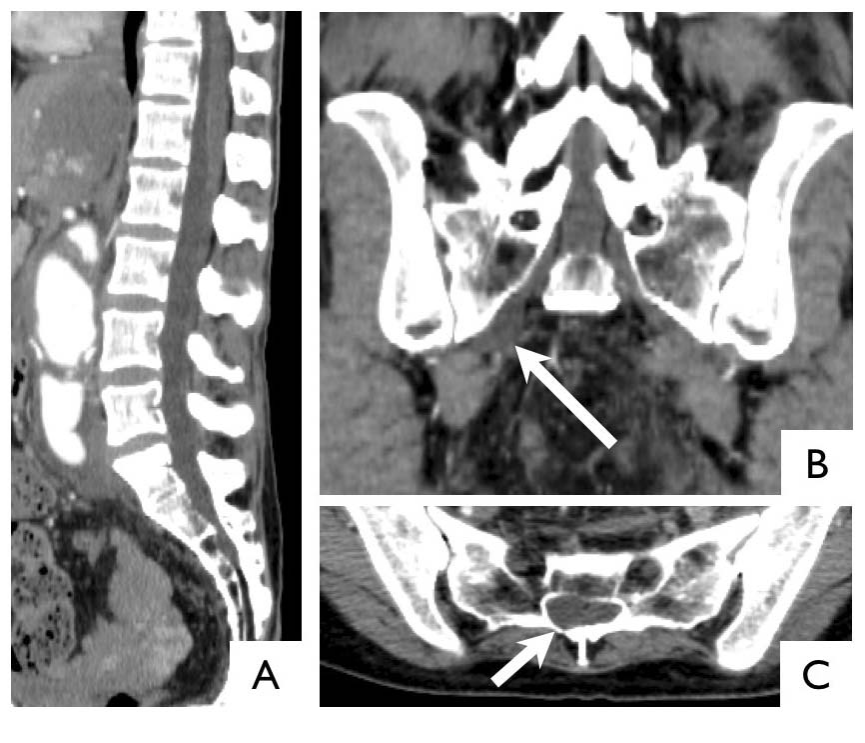
CT images of 46-year-old female with Loeys-Dietz syndrome. Sagittal image of the normal dura (A). Coronal image of right lateral meningocele (arrow) (B). Axial image at S1 shows asymmetric dilatation of the dura (arrow) (C). In this case, visual inspection could detect dural ectasia, but quantitative evaluation could not.

The results obtained with the qualitative method (Table 1) showed that 4 (40%) LDS and 16 (80%) MFS patients possessed DE, but none of the control subject did. DE were thus more frequently observed in LDS and MFS than in control (p = 0.0077 and <0.001, respectively). There was no anterior meningocele in LDS or control, but one in MFS, while one lateral meningocele was observed in LDS (Fig. 2) and five in MFS, but none in control.

According to findings obtained with method-1 (Table 1), 5 (50%) LDS and 15 (75%) MFS patients possessed DE, and none of the control subjects did. Higher prevalence of DE in LDS and MFS than in control was also observed with this diagnostic method (p = 0.0018 and <0.0001, respectively).

According to findings obtained with method-2 (Table 1), LDS (at L2) and MFS (at L5, S1) patients had a higher prevalence of DE than control. Seven (70%) LDS and 17 (85%) MFS showed the presence of DE and one (5%) control also showed DE. Most MFS patients diagnosed with method-2 had DE at S1or L5 while few showed DE at other levels. In addition, LDS patients showed a diffusely wide dura from L1 through to S1 except for L4. The values for DSR are also listed in Table 2.

**Table 2:** Mean DSR values.

	LDS (n = 10)	MFS (n = 20)	Control (n = 20)	CI*
L1	0.56±0.08	0.52±0.12	0.48±0.06	NS
L2	0.53±0.09	0.49±0.12	0.45±0.06	b, 0.0004−0.17
L3	0.48±0.08	0.45±0.09	0.42±0.06	NS
L4	0.45±0.07	0.47±0.11	0.44±0.02	NS
L5	0.53±0.10	0.56±0.15	0.43±0.08	c, 0.04−0.22
S1	0.60±0.11	0.88±0.54	0.40±0.07	c, 0.21−0.74

DSR, dural sac ratio; LDS, Loeys-Dietz syndrome; MFS, Marfan syndrome; DE, dural ectasia; NS, not significant; a, difference between LDS and MFS; b, difference between LDS and control; c, difference between MFS and control.

CI*, this column shows the confidence interval for significant differences.

## Discussion

DE usually occurs in connective tissue diseases, and was one of the major diagnostic criteria for MFS [6]. DE is no longer a critical criterion for MFS; however, DE still is important because it is used in the scoring system in the revised version [7]. While the importance of DE is well recognized in MFS, its prevalence in LDS is unknown. Although there is at present no standardized diagnostic method for DE in LDS, some qualitative [6], [8] and quantitative methods [9], [12], [13] have been used. In contrast to findings for MFS, only a few reports have dealt with the prevalence of DE in LDS [1], [10], [11]. Our study showed that LDS had a wider dural sac than control and the prevalence of DE in LDS was significantly higher than in control regardless of which diagnostic method was used. According to the findings obtained with the methods used in our study, the prevalence of DE in LDS varied from 40 to 70%. This prevalence is higher than the 16% reported by Rodrigues et al. [10]. Although their report does not mention any details of diagnostic criteria for DE, they also used CT images. The reason for the difference between the two studies is not clear. In this study, DE was positive in 75–85% for MFS and 0–5% for NML. In a clinical setting, the differential diagnosis of LDS from MFS is problematic. Although the difference in DE frequency observed using a qualitative method is significant (p = 0.04) between LDS and MFS, the differences between method-1 and method-2 are not significant. This result shows that only knowing DE frequency would not be helpful when attempting to distinguish LDS from MFS. Further examination is needed to determine the differences that exist between these two genetic vascular disorders in addition to DE frequency.

Some reports about DE in MFS have emphasized quantitative methods because cutoff values can be used more uniformly than with qualitative methods [12], [18]. However Lundby et al. reported that qualitative signs were very useful because 11.5% of their patients would not have been diagnosed with DE if lateral menigocele had not been adopted as a sign of DE [17]. Anterior and lateral meningocele has been identified as a strong qualitative indicator of DE in many studies [6], [9], [17]. We also propose the use of visual evaluation of DE, as presented in Fig. 2, because two (20%) LDS and three (6%) MFS patients were diagnosed by means of qualitative assessment although they were not diagnosed as such with method-1. As well as anterior or lateral meningocele, asymmetric dilatation such as scalloping of the vertebral body or widening of the neural foramina cannot be detected with qualitative evaluation in the mid-sagittal plain. In this study, the prevalence of anterior and lateral meningocele was lower than previously reported. This may be due to the lower contrast resolution of CT compared with MRI, which may lead to misdiagnosis of some meningoceles.

A higher prevalence of DSD at S1 than that at L4 (method-1) was used as a quantitative diagnostic method for DE [9] and resulted in assessment with high inter-observer agreement (κ = 0.77) [17]. This parameter can be easily and reliably measured in a routine clinical setting on CT and MRI, and previous studies have reported that it is also a useful marker for DE in [9], [18]. Our result showed that five (50%) LDS and 15 (75%) MFS patients showed DE with this quantitative method in contrast to control (0%), which is consistent with previously reported findings. While this parameter is thus a useful diagnostic tool, it can be somewhat problematic in that the difference in DSD at L4 and S1 is often very small. There were a total of seven cases among the LDS and MFS patients in our study with differences of less than 2 mm. It is also problematic that diffuse ectasis throughout the lumbosacral regions is not detected with this method.

DSR (method-2), which Oosterhof et al. firstly described in 2001 [12], has been used to assess DE in recent studies [13], [17]. The authors concluded that a combination of DSR above a given cutoff value at level L3 and S1 could be used to identify MFS with 95% sensitivity and 98% specificity. However, their method has been tested in later studies, but similar results have not been obtained [18], [19]. Habermann et al. found a sensitivity of 56% and a specificity of 65% with an optimal cutoff value of 0.51 at S1 [18]. They suggested that some of the differences in cutoff values and accuracy were secondary to the age differences of the subjects enrolled in the two studies. Cutoff values depend on the characteristics of the control group such as age, ethnos, and diagnostic modality (i.e. CT or MRI). The main purpose of the cutoff values adopted for our study (means+2 SD of controls) was therefore to reduce the influence of such dependence. The DSRs reported by Lundby were 0.45, 0.43, 0.42, and 0.41 at L3, 4, 5, and S1, respectively [17], and those reported by Oosterhof were 0.48, 0.40, 0.35, 0.34, 0.32, and 0.35 at L1, 2, 3, 4, 5 and S1, respectively, [12]. These results were thus not so different from ours. By using our cutoff values, we identified DE in seven (70%) LDS and 17 (85%) MFS patients. Because most of LDS and MFS patients showed DE at S1, the prevalence of DE obtained with method-2 was equivalent to that obtained with method-1 in our study. As mentioned before, method-1 cannot identify an abnormality when the dilatation is diffuse (i.e., L4 was dilatated as well as S1). As seen in Table 2, LDS patients, in contrast to MFS patients, feature a diffuse dilatation, so that the use of cutoff values may help diagnose the dilatation of DE correctly even in LDS patients. In addition, the finding of the diffuse distribution of DE may help to identify LDS and distinguish it from MFS, although the difference between LDS and MFS was not significant in our study.

In a recent report, Soylen et al. reported 100% sensitivity and 94.7% specificity for a novel quantitative method using MRI images [13]. They adopted the value calculated by multiplying longitudinal diameter by wide diameter of the dura in axial plane for each level. They compared their results with those of Ahn and Oosterhof and found that sensitivity was equivalent for the three methods but their specificity was superior to Oosterhof’s. We also adopted their qualitative method using CT images (unpublished data). While our results showed that many LDS and MFS patients had DE, the prevalence was very similar to that assessed with method-2. Soylen et al.’s method has a good diagnostic performance, but the measurements are rather time-consuming, so that the method proposed by Ahn or Oosterhof may be satisfactory for clinical settings.

Compared with CT, MRI is superior in quality of contrast resolution, especially in visualization of soft tissue. Therefore, most previous studies have used MRI imaging for the evaluation of DE [9], [12], [13]. However, thin slice data can now be obtained easily with CT, and MPR images derived form CT make it possible to analyze objects easily from any plane. In our institution, we usually reconstruct CT images of 2 mm slice thickness and diagnose them using a PACS viewer. Under these circumstances, we can reconstruct MPR images and analyze them within 5 min per patient. We applied MRI criteria for DE to our CT measurements. This is because we believed that these MRI criteria could be used with current CT images. As explained above, lateral and anterior meningocele constitutes an important finding of DE [17]. Reconstructed MPR CT images can also be used for the evaluation of both lateral and anterior meningocele. The fact that T2-weighted images on MRI are superior for detecting abnormalities with a water component makes the detection of lateral and anterior meningocele using MRI feasible. Moreover, careful reading of axial and MPR images of CT is sure to improve the detectability of anterior and lateral meningocele.

In this study, LDS showed a higher prevalence of DE than controls. If the presence of DE turns out to be a useful finding for differentiating LDS from controls, it may well become one of the diagnostic criteria. However, further study is needed to evaluate its diagnostic performance and feasibility.

Our study has certain limitations. First, while the number of patients in our study was very small, it is comparable with the numbers used in other studies. LDS patients in this study were consecutively recruited and represented the maximum number of this type of patient at that time. However, our study did not include patients aged <20 because there were no patients of that age in our hospital. This may have caused a patient selection bias, thereby influencing our results. Our results for MFS and controls were comparable with those of a study comprising a large number of MFS patients [17]. For LDS, on the other hand, further studies with a large number of subjects is necessary. Second, because to some extent DE develops with age, some patients may be without DE in spite of gene abnormalities. In this respect, we did not calculate either the sensitivity or specificity for the evaluation of the diagnostic performance against the genetic diagnosis as a reference standard. Of course, the prevalence of DE may fluctuate to some extent depending on the characteristics of the groups.

## Conclusions

LDS as well as MFS showed a higher prevalence of DE than controls. The prevalence of DE in LDS varied from 40 to 70% depending on different qualitative and quantitative methods. These findings indicate that DE has the potential to become a diagnostic criterion for LDS.
